# Do Children With Autism Spectrum Disorders Understand Pantomimic Events?

**DOI:** 10.3389/fpsyg.2019.01382

**Published:** 2019-06-18

**Authors:** Ines Adornetti, Francesco Ferretti, Alessandra Chiera, Slawomir Wacewicz, Przemysław Żywiczyński, Valentina Deriu, Andrea Marini, Rita Magni, Laura Casula, Stefano Vicari, Giovanni Valeri

**Affiliations:** ^1^ Cosmic Laboratory, Department of Philosophy, Communication and Performing Arts, Roma Tre University, Rome, Italy; ^2^ Department of English, Center for Language Evolution Studies CLES, Nicolaus Copernicus University, Torun, Poland; ^3^ Department of Languages and Literatures, Communication, Education and Society, University of Udine, Udine, Italy; ^4^ Scientific Institute, IRCCS E. Medea, San Vito al Tagliamento, Pordenone, Italy; ^5^ Child and Adolescent Neuropsychiatry Unit, Department of Neuroscience, Bambino Gesù Children’s Hospital, IRCCS, Rome, Italy

**Keywords:** comprehension of actions, autism, gesture, mirror neurons, motor representation, pantomime

## Abstract

Impairments of motor representation of actions have been reported as a core component of autism spectrum disorders (ASD). Individuals with ASD have difficulties in a number of functions such as assuming anticipatory postures, imitating body movements, producing and understanding gestures, and recognizing motor intentions. Such cognitive-motor abilities are all involved in pantomime. However, the available evidence on the production and comprehension of pantomime in individuals with ASD is still inconclusive. The current investigation assessed pantomime comprehension in 40 children with high-functioning ASD and 40 children with typical development balanced for age, IQ, level of formal education, and cognitive profile. The participants were asked to watch video recordings of pantomimes representing simple transitive events enacted by actors and match them to the corresponding pictorial representations. Such pantomimes were delivered in two conditions with different levels of information content (i.e., lean or rich). The two groups of children performed similarly on these tasks. Nonetheless, children with ASD who were administered the pantomimes in the lean condition performed worse than participants who were administered the informatively richer pantomimes. The methodological implications for interpretation of previous findings and future studies are discussed.

## Introduction

### Autism Spectrum Disorders and Praxic Competence

Autism spectrum disorders (ASD) are characterized by two core symptoms: (1) the presence of restricted, repetitive patterns of behavior, interests, or activities, and (2) persistent deficits in social communication and interaction [DSM-5 American Psychiatric Association (APA), 2013]. The fifth edition of the Diagnostic and Statistical Manual of Mental Disorders (DSM-5 American Psychiatric Association [APA], 2013) describes a cluster of symptoms that affect the social domain by including difficulties in nonverbal communicative behavior, abnormalities in body language, or deficits in gestural understanding and use. Such problems have already been highlighted by Kanner (1943). In his seminal paper, he reported that infants with the disorder were unable to make anticipatory motor adjustments, e.g., they failed “*to assume at any time an anticipatory posture* preparatory for being picked up” (Kanner, 1943, p. 242). Subsequent investigations confirmed that persons with ASD exhibit impairments in the motor domain; some scholars have even suggested that such impairments might represent a core component of autism (e.g., De Meyer et al., 1972; Jones and Prior, 1985; Rogers et al., 1996; Smith and Bryson, 2007; Cossu et al., 2012; Gizzonio et al., 2015; Bo et al., 2016).

Motor impairments in ASD are present in two different dimensions: motor control and praxic performance (Gizzonio et al., 2015). The deficits in basic motor control affect different functions such as gait, posture, and coordination. For instance, Lane et al. (2012) showed that in the first year of life, young children with ASD may have delays in supine, prone, and sitting skills. Gernsbacher et al. (2008) reported that toddlers with ASD might experience difficulties in reaching, clapping, and pointing. Studies have also showed that school-aged children with ASD exhibit motor delays in tasks such as graphomotor control, manual dexterity, and balance (Miyahara et al., 1997; Hilton et al., 2007; Provost et al., 2007; Fuentes et al., 2009). Such difficulties in the basic level of motor control often persist into childhood and may adversely affect the development of a number of other abilities, such as drawing, speaking, and playing (Jansiewicz et al., 2006).

Particularly relevant to the aims of the current study are the deficits affecting praxis, i.e., the ability to perform skilled movements (Smith and Bryson, 2007) that cannot be ascribed to basic coordination impairments (Gibbs et al., 2007; Gizzonio et al., 2015). Common deficits in praxis include recognition of motor intentions (action goals) and production and comprehension of gestures (Rogers and Pennington, 1991; Rogers et al., 1996; Mostofsky et al., 2006; Hamilton et al., 2007; Knaus et al., 2017). In the experimental literature on ASD, such symptoms have been mainly investigated in relation to action imitation abilities (e.g., Rogers et al., 1996; Schunke et al., 2016; Xavier et al., 2018; for a discussion: Rogers and Pennington, 1991; Williams et al., 2004; Vivanti and Hamilton, 2014). Research on imitation in autism may be relevant not only to the investigation of imitative skills as such, but also to the investigation of the pathogenesis of ASD (Rogers and Pennington, 1991; Rogers and Williams, 2006; Cattaneo et al., 2007; Cossu et al., 2012). Indeed, Rogers and Pennington (1991) proposed that impairments in imitation in ASD might be related to a deficit in forming and coordinating specific social representations of the self and the others. Therefore, the social and communicative problems frequently reported in persons with ASD might at least partially be related to more basic deficits in motor imitation: early imitation provides information about other individuals, in a way that may play a pivotal role in the development of a sense of other minds (Meltzoff and Gopnik, 1993). According to several studies, a dysfunction of the mirror neuron mechanism (MNM) might explain both poor performance on imitation and impaired social and communicative abilities in individuals with ASD (Williams et al., 2001; Oberman and Ramachandran, 2007). The MNM involves a network of regions including the inferior parietal and inferior frontal cortex, which leads to the conclusion that they provide the neural substrate for matching action perception and execution. Such a network is activated both when an individual performs an action and when (s)he observes another person performing the same action (di Pellegrino et al., 1992; Rizzolatti and Craighero, 2004), thus facilitating the mirroring of one’s own and others’ actions. In this way, the MNM significantly contributes to the ability to infer the goals and intentions of others (Gallese and Goldam, 1998; Hamilton and Grafton, 2006), which involves both recognition (action observation and comprehension) and imitation involved in gestural representations (Buccino et al., 2004; Corballis, 2010).

In a seminal study, De Meyer et al. (1972) reported that individuals with ASD had difficulties in several tasks involving body imitation, motor-object imitation, and spontaneous object use. Interestingly, such preliminary findings were subsequently supported by other studies (Rogers et al., 1996; Zwaigenbaum et al., 2005; Smith and Bryson, 2007). For example, Zwaigenbaum et al. (2005) reported difficulties in the imitation of actions on objects in 1-year-old infants with probable ASD. Other studies showed that children with ASD have difficulty in imitating so-called “meaningful gestures,” i.e., conventional gestures such as the “thumbs up” gesture (Beadle-Brown, 2004; Dewey et al., 2007; Smith and Bryson, 2007). Research on adolescents and adults with ASD highlighted difficulties also in the imitation of nonmeaningful gestures, i.e., actions that do not convey a specific meaning (e.g., Rogers et al., 1996; Vivanti et al., 2008). Yet, findings are still far from conclusive (see Vivanti and Hamilton, 2014), as other investigations did not support the hypothesis of a core imitation deficit in ASD. For example, in Hamilton et al. (2007), children with ASD performed better than children with typical development on tasks of gesture recognition. Of note, a high heterogeneity in imitation performance in individuals with ASD is not an uncommon finding (e.g., Beadle-Brown and Whiten, 2004; Bird et al., 2007; Press et al., 2010; Rogers et al., 2010; Vivanti et al., 2011; Salowitz et al., 2013), suggesting that imitation difficulties can be observed in many but not all individuals diagnosed with ASD.

### Pantomime in Autism Spectrum Disorders

Studies focusing on the ability to produce and understand pantomimed actions in individuals with ASD reported conflicting results (Rogers et al., 1996; Ham et al., 2011; Cossu et al., 2012; Gizzonio et al., 2015). With regard to *production*, pantomime has been mainly analyzed in reference to simple actions, either transitive (actions requiring interaction with objects, such as cutting something with scissors) or intransitive (symbolic/representational actions, such as waving good-bye) across two conditions: tasks with pantomimed actions elicited by pictures and tasks with pantomimed actions elicited by a verbal command. In a study by Gizzonio et al. (2015), children were required to show both the use of a specific object (e.g., a glass of water) and execute representational gestures (e.g., summoning somebody). Smith and Bryson (2007) and Cossu et al. (2012) asked children to generate pantomimic actions in response to pictures of objects (e.g., they had to pantomime the act of cutting when the picture they saw represented the scissors). The results of these studies showed decreased performance in pantomimic action execution in children with ASD in both conditions. These results support the hypothesis of a deficit in the production of pantomime in ASD when imitation is elicited by either pictures or verbal commands. However, experiments assessing pantomime *comprehension* led to contrasting results. For example, Cossu et al. (2012) reported that children with ASD had difficulties both in the visual and in the oral comprehension of pantomimes (see also Ham et al., 2011). But Smith and Bryson (2007) showed that children and adolescents with ASD had no difficulty in recognizing pantomimed actions and understanding their meaning.

### Pantomime: Narrow Versus Broad

The conflicting results observed on tasks assessing pantomime comprehension in ASD may lead to two different hypotheses. The first of these is that problems in production, frequently reported in ASD children, stem from an underlying motor representation deficit. Conversely, a lack of differences between individuals with ASD and individuals with typical development on pantomime comprehension tasks can be taken to suggest that the production deficits in ASD are specific to production and that more general impairments of motor representation should not be considered as a core feature of ASD.

In order to further explore these two possibilities, the current study was designed to assess the ability to understand pantomimic events in a large cohort of children with ASD aged 7–11. We adopted a richer definition of pantomime than the ones found in previous studies. “Pantomime” is, indeed, a complex term whose technical meanings vary across disciplines (some with centuries if not millennia of tradition – see Żywiczyński et al., 2018). Many of the previously cited studies used a “narrow” interpretation of pantomime, i.e., pantomimes that are based on the execution of a relevant motor sequence in the absence of an instrumental goal or of its object for transitive actions. A narrow definition of pantomime is also typical of experiments in cognitive neuroscience (e.g., Rothi et al., 1985; Dumont et al., 1999), in particular in research on apraxia, where pantomime is used for pretend tool use (e.g., the use of an imaginary hammer or paintbrush). This is sometimes distinguished from gestures representing intransitive actions (e.g., hitchhiking), which are more dependent on social and cultural information as well as on lexical knowledge (for a discussion on this topic, see Bartolo et al., 2003).

In contrast, a “broad” definition of pantomime describes it as a nonverbal, mimetic, and non-conventionalized means of communication in which events are represented by coordinated movements of the whole body (Żywiczyński et al., 2018). Such a definition of pantomime is more communicatively natural, as it relies on entire events, and each pantomime functions as a self-contained communicative act. It is also considerably richer with regard to content and more complex than the production and comprehension of most representational gestures, which tend to convey individual concepts rather than events. Most importantly, “true” pantomime is a type of representational gesture that involves:

1. *enactment* – body-to-body mapping, rather than body-to-object mapping (e.g., as in pretend tool use; Gärdenfors, 2017, see also “character viewpoint” vs. “object view point”: Beattie and Shovelton, 2002; hand-as-hand vs. hand-as-object gesture: Marentette et al., 2016; or egocentric vs. allocentric: Brown et al., 2019);
2. *whole-body movement*, rather than predominantly manual (Gärdenfors, 2017);
3. *robust iconicity* – iconicity constitutes its dominant *semiotic ground* (Jakobson, 1971/1965; Peirce, 1992; Ahlner and Zlatev, 2010), and it is “primary iconicity,” i.e., sufficient to infer the meaning of the pantomime. In this respect, pantomime is unlike many other representational gestures, whose iconicity is “secondary,” that is the iconic ground alone does not suffice to infer the meaning, and the iconic relation can only be recognized once the meaning is known (see Sonesson, 1997);
4. *reference to events* rather than individual actions or objects (minimally an event involves a definable Agent; in our study, we focused on transitive events; see Zlatev et al., 2017 for the application of the same paradigm).

This broader definition of pantomime involves all of the main features of the motor representation of actions (i.e., postures, body movements, gestures, and motor intentions) and requires their integration with higher level cognitive skills responsible for the interpretation of event structures. For example, general representations of events have been suggested to rest on working memory capacity. The literature on “event cognition” (e.g., Radvansky and Zacks, 2014) shows that representing a single event requires people to keep track of the various aspects involved in that event and to integrate those aspects with information from both the environment and their world knowledge. Working memory enables the construction of event models by making it possible to maintain such pieces of information that are relevant for one’s own current activity. This is supported by evidence on older adults who show a decline in working memory capacity: they have been reported to keep track of fewer pieces of information and to construct less complete event models (e.g., Craik and Byrd, 1982).

Overall, the broad definition of pantomime makes it a more sensitive diagnostic tool for detecting deficits in the comprehension of meaningful communicative actions. In the light of these considerations, the current study aims at assessing: (1) whether impairments of comprehension of pantomimic events could be identified in children with high-functioning ASD; (2) whether the content of pantomimes – lean versus rich – would influence children’s performance.

## Materials and Methods

### Participants

Two groups of children aged between 7 and 11, matched on chronological age, level of formal education, and IQ level (see Table 1), took part in this study. The first one consisted of 40 children with high-functioning ASD recruited at Bambino Gesù Children’s Hospital in Rome, Italy. The Raven’s Coloured Progressive Matrices [(Raven, 1938); Italian standardization: Belacchi et al., 2008] were employed to assess their IQ, which turned out to be within the normal range. The diagnosis of ASD was established by the neuropsychologists of Bambino Gesù Children’s Hospital and was based on clinical observation in compliance with the Diagnostic and Statistical Manual of Mental Disorders-V criteria [DSM-5 American Psychiatric Association (APA), 2013]. The severity of autistic symptomatology was ascertained through the administration of the Autism Diagnostic Observation Schedule 2nd edition – ADOS-2 by Lord et al. (2013). Overall, the group of participants with ASD had a mean severity score of 5.90 with a standard deviation of 1.42 ranging from 3 to 8.

**Table 1:** General data of the two groups of participants.

	ASD (*n* = 40)	TD (*n* = 40)
Age	9.39 (0.99), range: 7–11.02	9.42 (1.00), range: 7.1–11.7
Education	1st–5th grade	1st–5th grade
Gender distribution	Males = 31 (77.5%)	Males = 25 (62.5%)
IQ level	111.00 (11.94), range: 90–130	111.25 (12.02), range: 90–130
ADOS-2 severity Index	5.90 (1.42), range = 3–8	

Forty children with typical development (TD) formed the control group. TD children obtained scores within the normal range on the Raven’s Progressive Matrices (Raven, 1938; Italian standardization: Belacchi et al., 2008) aimed at assessing their levels of nonverbal intelligence. In a preliminary interview, their teachers confirmed that they had normal cognitive development, as well as average school performance. According to school records and parents’ reports, none of them had a known history of psychiatric or neurological disorders, learning disabilities, hearing or visual loss.

In order to obtain a general cognitive profile of the two groups, both children with ASD and TD were administered tasks assessing their verbal short-term and working memory, namely the Non-Word Repetition subtest of the Prove di Memoria e Apprendimento per l’Età Evolutiva (PROMEA, Vicari, 2007) and the Forward and Backward Digit Span’s subtests of the Wechsler Scales (Wechsler, 1993).

The Bambino Gesù Children’s Hospital committee approved this study. Parents signed the consent form for the participation of their children to the study and for the treatment of the data.

### Methods

The children with of ASD were tested at Bambino Gesù Children’s Hospital in Rome. The children of the control group were tested individually at school. Participants were administered tasks assessing their phonological short-term memory and working memory, attention skills, and theory of mind. Finally, participants were administered a task assessing their comprehension of pantomimic events.

#### Assessment of Phonological Short-Term and Working Memory

The Non-Word Repetition Task of the PROMEA (Vicari, 2007) and the Digit Span Forward and Backward subtests of the Wechsler (1993) Scales were employed to assess phonological short-term and working memory. This was done with two goals in mind: firstly, to obtain a general cognitive profile of the two groups of participants; secondly, to control for a potential involvement of working memory, since it has been hypothesized (Bartolo et al., 2003; Villarreal et al., 2008) that such a component of memory has an important role in the processing of pantomimed actions, as it keeps the information online while generating a mental representation of the pantomimically enacted action.

In the Non-Word Repetition task, the child had to repeat 40 non-words read by the examiner aloud while concealing the movements of the lips. For each correct answer, the child received 1 point, for a maximum of 40 correct repetitions.

In the Digit Span forward task, the child was required to repeat in the same order the sequences of digits pronounced by the examiner. The length of digits started with a sequence of three numbers and gradually increased to a sequence of a maximum of nine items. The number of lists correctly repeated by the child represents the Digit Span forward score. In the Digit Span backward task, the child was asked to repeat each sequence in the reverse order. In this case, the longest list contained eight items.

#### Assessment of Attention Skills

As praxis disorders in ASD may stem from attention deficits (Press et al., 2010), the selective attention and sustained attention of the participants were evaluated by administering the Modified Little Bells’ test (Biancardi and Stoppa, 1997). The children were presented with four different sheets, each containing a series of little bells and additional drawings of animate and inanimate objects (e.g., houses, trees, horses, fishes, etc.). The children were asked to mark the little bells in the sheet as quickly as possible over a 2-min time period for each sheet. During the first 30 s, the children were instructed to mark the bells using a red pen; for the remaining 90 s, they were asked to use a blue pen. The participants were not aware of how much time they had, nor how many sheets would be presented to them or the number of bells on each sheet.

Two scores were obtained from the administration of this test: the rapidity score and the accuracy score. The former was obtained by summing up the total number of bells found per sheet in the first 30 s: such score corresponded to the children’s selective attention. The accuracy score was calculated by summing up the total number of bells found on all four sheets after the 2-min: this score corresponded to the children’s sustained attention.

#### Assessment of Theory of Mind

As impairments in action understanding in ASD may be also related to a deficit in social cognition (Rogers and Pennington, 1991), we assessed the participants’ ability to understand other persons’ perspectives and to recognize emotions related to different situations. To this aim, the children were administered the Theory of Mind-part II subtest from the NEPSY-II (Korkman et al., 2007). In the Theory of Mind-part II task, the children were shown nine pictures representing a girl depicted from behind in several contexts (e.g., arguing with a friend, on a roller coaster, playing with cats). They were then presented with four pictures of emotional facial expressions, and were asked to identify the one best matching the girl’s expression in that specific situation. The first item was used as a trial to allow children to get acquainted with the test. Each correct answer was assigned 1 point for a maximum score of 8 points.

#### Pantomime Comprehension Task

**Figure 1:**
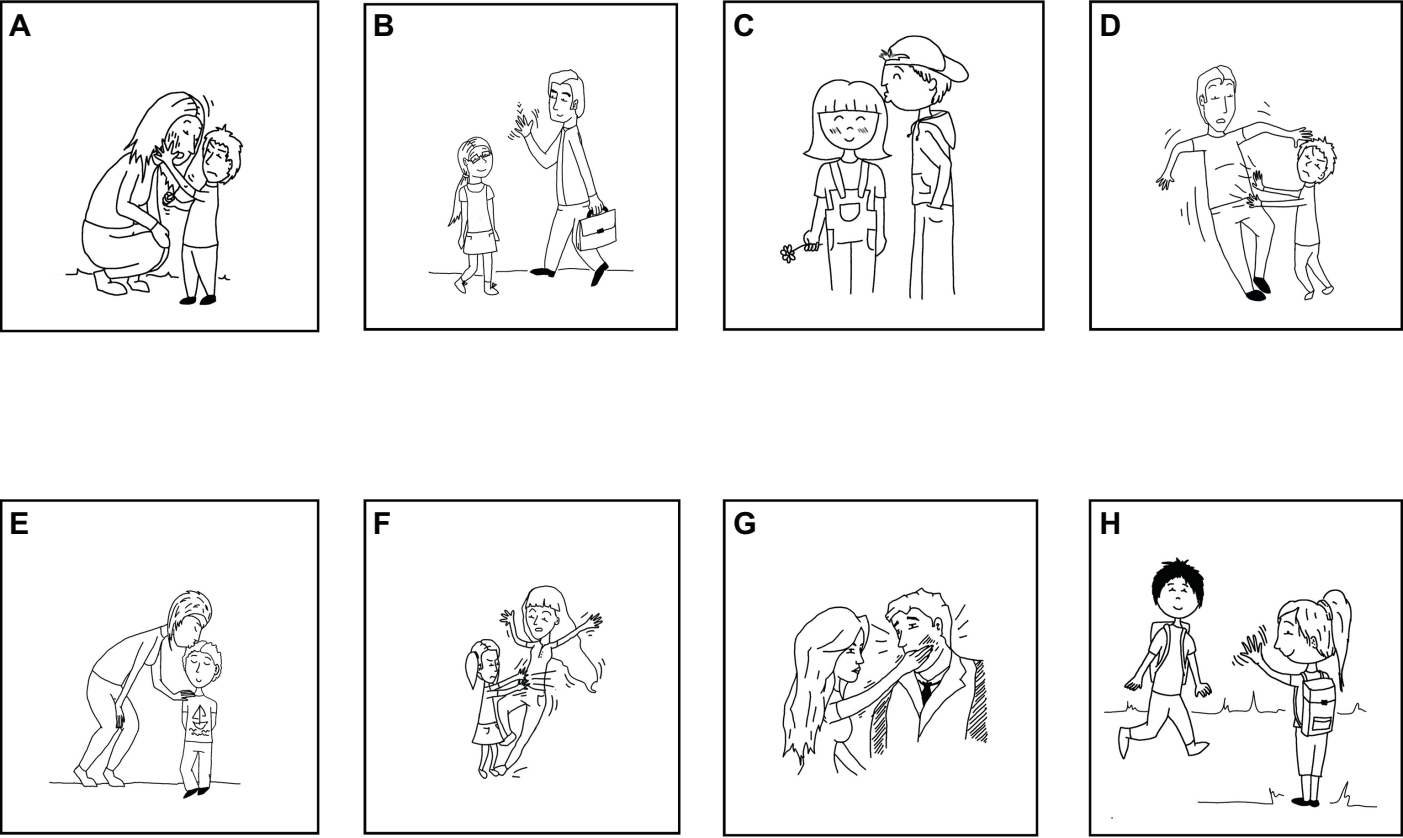
Response sheet used in the picture-matching task aimed at assessing the comprehension of pantomime. Events from **(A)** to **(H)** were obtained combining three elements: an Action (i.e., kiss, wave, slap, and push); an Agent (i.e., man, woman, boy, and girl); a Patient/Beneficiary (i.e., man, woman, boy, and girl). Adapted from Zlatev et al. (2017).

In the pantomime comprehension task (adapted from Zlatev et al., 2017), the children were asked to watch video clips displayed on a PC monitor. Each video clip showed one amateur actor who enacted – that is, presented by means of expressive whole-body pantomime – a simple transitive event. Each event was decomposable into three elements: an Action (i.e., kiss, wave, slap, or push); an Agent (i.e., man, woman, boy, or girl); and a Patient/Beneficiary (i.e., man, woman, boy, or girl). Each child saw nine video clips, of which the first one served as a trial to allow the children to get acquainted with the test, and the remaining eight constituted the test session. In the test session, each video clip was preceded by a 4-s fade-in screen showing the clip number. The individual clips (e.g., a boy kissing a girl, a woman slapping a man, a man waving at a girl, etc.) were played one after another. After each item, the playback was stopped, and the child was asked to indicate on a response sheet with drawings depicting different events, which of the eight drawings best matched the event they had just watched (see Figure 1). No time constraints were imposed. Each correct answer was assigned 1 point for a maximum score of 8 points.

This task was administered in two conditions: an informatively lean “half-event” condition, and an informatively rich “full event” one. In the informatively lean (IL) condition, the actor pantomimed only the agent of the event. For example, to represent the event D (see Figure 1) with a boy pushing a man, the actor pantomimed only the action performed by the boy. In the informatively rich (IR) condition, the actor pantomimically played the part of both participants, i.e., the agent and the patient/beneficiary of the action. In this case, to perform the event D (Figure 1), the actor first pantomimed the boy pushing the man and, immediately afterward, the man jumping back.

The two groups of participants (i.e., children with ASD and those with TD) were divided into four subgroups: children with ASD who were shown the pantomimes in the informatively lean condition (ASD-IL); children with ASD who were shown the pantomimes in the informatively rich condition (ASD-IR); children with typical development who were shown the pantomimes in the informatively lean condition (TD-IL); children with typical development who were shown the pantomimes in the informatively rich condition (TD-IR). Each subgroup was formed by 20 participants. The stimulus video file representing the pantomime in the informatively lean condition had a duration of 88 s (fade-in screens included). The stimulus video file representing the pantomime in the informatively rich condition had a duration of 145 s (fade-in screens included).

## Results

### Analysis of Phonological Short-Term and Working Memory, Attention Skills, and Theory of Mind

The group-related differences on the assessment of the children’s cognitive skills were analyzed with a series of *t*-tests with group (i.e., ASD vs. TD) as fixed factor and the six cognitive measures (i.e., the scores on Non-Word Repetition Task of the PROMEA; the scores on the Forward and Backward Digit Span subtests of the WISC; selective attention – rapidity score; sustained attention – accuracy score; theory of mind score) as dependent variables. As shown in Table 2, the two groups did not differ on measures assessing their general cognitive skills. Indeed, children with ASD and children with TD had similar performance on tasks assessing phonological short-term and working memory: the Non-Word Repetition Task [(*t*_78_ = 0.166); *p* = 0.869] and both Forward (*t*_78_ = −1.901; *p* < 0.061) and Backward Digit Span [(*t*_78_ = 0.66); *p* = 0.948] subtests of the WISC. Furthermore, they obtained similar results on tasks assessing Theory of Mind [(*t*_78_ = −0.734); *p* = 0.465] and attention skills {rapidity score [(*t*_78_ = −0.281); *p* = 0.779] and accuracy score [(*t*_78_ = −0.968); *p* = 0.336]}.

**Table 2:** Cognitive profile of the two groups of participants.

	ASD *M* (SD) [min–max]	TD *M* (SD) [min–max]
Non-word repetition	34.45 (4.11) [22–40]	34.30 (3.96) [25–40]
Forward digit span	7.33 (1.16) [5–10]	8.00 (1.92) [5–13]
Backward digit span	5.03 (1.81) [0–8]	5.00 (1.58) [3–9]
Selective attention – rapidity score	48.38 (13.51) [24–78]	49.13 (10.11) [26–71]
Sustained attention – accuracy score	115.63 (19.06) [57–138]	110.93 (24.09) [45–139]
Theory of mind	6.23 (1.14) [3–8]	6.40 (0.98) [4–8]

*Data are expressed as means, standard deviations, and ranges. Legend: ASD, (children with) autism spectrum disorders; TD, (children with) typical development*.

### Analysis of Comprehension of Pantomimic Events

Potential group-related differences on the pantomime comprehension task were explored with one ANOVA with group (i.e., ASD vs. TD) as fixed factor and the pantomime comprehension score as the dependent variable. The children with ASD had a mean score of 4.35 with a standard deviation of 1.73 ranging from 1 to 8; children with TD had a mean score of 5.08 with a standard deviation of 1.99 ranging from 1 to 8, and the analysis did not reveal any significant difference between the two groups on this general measure [*F*(1, 76) = 0.507, *p* = 0.479].

To explore the possibility that the specific condition (i.e., IL vs. IR) in which pantomimic events were presented to the participants influenced the comprehension of the task, the groups of children with ASD and TD were split into four subgroups: children with ASD who saw the pantomimes in the informatively lean condition (ASD-IL); children with ASD who saw them in the informatively rich condition (ASD-IR); children with typical development who saw the pantomimes in an informatively lean condition (TD-IL); children with typical development who saw them in an informatively rich condition (TD-IR). The four subgroups had comparable chronological age, IQ level, and cognitive profile, but differed in the performance on the Forward Digit Span subtest of the WISC [*F*(3, 79) = 2.944, *p* < 0.038] (see Table 3). A series of Tukey’s *post hoc* tests showed that the children with ASD who saw the pantomimes in the informatively lean condition (ASD-IL) had lower scores on the Forward Digit Span Task than those with ASD who were administered them in the informatively rich condition (ASD-IR) (*p* < 0.049).

**Table 3:** General data and cognitive profile of four subgroups.

	ASD-IL (*n* = 20) *M* (SD) [min–max]	ASD-IR (*n* = 20) *M* (SD) [min–max]	TD-IL (*n* = 20) *M* (SD) [min–max]	TD-IR (*n* = 20) *M* (SD) [min–max]
Age	9.42 (1.05) [7.07**–**11.03]	9.36 (0.96) [7.07**–**11]	9.35 (1.03) [7.02**–**11.03]	9.50 (0.99) [8.00**–**11.03]
IQ	107.50 (11.18) [90**–**130]	114.50 (11.91) [100**–**130]	114.50 (12.34) [90**–**130]	108.00 (11.05) [90**–**130]
ADOS-2 severity Index	6.00 (1.37) [3**–**8]	5.80 (1.50) [3**–**8]		
Non-word repetition	33.95 (4.71) [22**–**40]	34.95 (3.45) [25**–**39]	35.30 (3.70) [28**–**40]	33.30 (4.05) [25**–**39]
Forward digit span*	7.25 (1.29) [5**–**10]	7.40 (1.04) [5**–**9]	7.45 (1.76) [5**–**11]	8.55 (1.95) [6**–**13]
Backward digit span	4.90 (2.10) [0**–**8]	5.15 (1.53) [2**–**8]	4.85 (1.72) [3**–**9]	5.15 (1.46) [3**–**9]
Selective attention – rapidity score	49.60 (13.58) [27**–**78]	47.15 (13.68) [24**–**73]	45.65 (7.22) [26**–**61]	52.60 (11.50) [25**–**71]
Sustained attention – accuracy score	117.85 (16.06) [83**–**138]	113.40 (21.85) [57**–**138]	108.80 (24.68) [45**–**139]	113.05 (23.92) [55**–**136]
Theory of mind	6.30 (1.17) [3**–**8]	6.15 (1.13) [4**–**8]	6.50 (1.00) [4**–**8]	6.30 (0.97) [5**–**8]

Data are expressed as means, standard deviations, and ranges. Legend: ASD-IL (children with) autism spectrum disorders who were administered the pantomimes in the informatively lean condition; ASD-IR (children with) autism spectrum disorders who were administered the pantomimes in the informatively rich condition; TD-IL (children with) typical development who were administered the pantomimes in the informatively lean condition; TD-IR (children with) typical development who were administered the pantomimes in the informatively rich condition. Asterisks (*) show when group-related differences were significant.

As the Forward Digit Span scores and the pantomime score were positively correlated (*r* = 0.287; *p* < 0.010), the group-related differences on measures assessing children’s comprehension of pantomimes in the two conditions were analyzed by performing a series of ANCOVAs with subgroups as fixed factors (ASD-IL; ASD-IR; TD-IL; TD-IR), the pantomime scores as dependent variables, and the participants’ performance on the Forward Digit Span subtest of the WISC as a covariate. The analyses revealed the presence of a group effect on the comprehension of pantomimic events [*F*(3, 75) = 4.598, *p* < 0.005]. Namely, a series of Tukey’s *post hoc* analyses showed that children with ASD who saw the pantomimes in the informatively lean condition obtained lower scores than both children with ASD and children with TD who saw them in the informatively rich condition (*p* < 0.034 and *p* < 0.001, respectively). Similarly, children with typical development who saw pantomimes in the informatively lean condition obtained lower scores than children with TD who saw them in the informatively rich condition (*p* < 0.008) (see Table 4).

**Table 4:** Performance of the four subgroups on the task assessing comprehension of pantomimic events.

	ASD-IL	ASD-IR	TD-IL	TD-IR	*Post hoc*
Comprehension of pantomimic events	3.75 (1.58) [1–6]	4.95 (1.70) [2–8]	4.20 (1.98) [1–8]	5.92 (1.60) [3–8]	ASD-IL < ASD-IR ASD-IL < TD-IR TD-IL < TD-IR

*Data are expressed as means, standard deviations, and ranges. Legend: ASD-IL (children with) autism spectrum disorders who were administered the pantomimes in the informatively lean condition; ASD-IR (children with) autism spectrum disorders who were administered the pantomimes in the informatively rich condition; TD-IL (children with) typical development who were administered the pantomimes in the informatively lean condition; TD-IR (children with) typical development who were administered the pantomimes in the informatively rich condition*.

## Discussion

The current study analyzed comprehension of pantomimed events – a specific aspect of the motor representation of actions – in a group of school-aged children with high-functioning ASD. Children with different severity levels of autistic symptomatology participated in the study, which allowed us to avoid a potential severity bias. The performance of the group of children with ASD was compared to that of a group of children with typical development matched on age, level of formal education, IQ, and cognitive profile. The comprehension of pantomimes was assessed by administering a task in two conditions differing in information richness: the informatively lean “half event” condition and the informatively rich “full event” condition. The participants with ASD obtained lower scores than children with TD on the task assessing pantomime comprehension independently of the condition. However, this difference was not statistically significant. When the two groups were divided into the four subgroups to assess the role of the two conditions (i.e., IL vs. IR), the subgroup of children with ASD who saw the informatively lean video clips performed worse than both the children with ASD and TD who saw the informatively rich ones; the subgroup of children with TD who saw informatively lean video clips obtained lower scores than the subgroup of TD children who saw the informatively rich ones. This suggests that pantomimic events presented in the informatively lean condition were more difficult to understand than those presented in the informatively rich condition for both the children with ASD and with typical development. Overall, these results have important theoretical and methodological implications.

The similar performance of children with ASD and with TD on the comprehension of pantomime irrespective of the two conditions was an unexpected finding. Indeed, it is at odds with the results obtained by Ham et al. (2011) and Cossu et al. (2012). Ham et al. (2011) investigated the ability to imitate and recognize three types of representational gestures (actions involving objects, actions with symbolic meaning without objects and pantomime of object use) in children with ASD aged 7–15. They found poorer performance in the ASD group on all recognition and imitation tasks. Cossu et al. (2012) examined three domains of motor representation – imitation of actions, production and comprehension of pantomime. Their results showed that children with ASD performed significantly worse than control groups (children with the same chronological age and children of chronological younger age) on the three conditions. According to Cossu et al. (2012), these results support the hypothesis of an impairment of both production and comprehension of actions in ASD and suggest an early damage to the MNM responsible for these deficits.

In line with other studies that investigated both gesture imitation and recognition (Smith and Bryson, 1998; Hamilton et al., 2007) and pantomimed object use (Smith and Bryson, 2007), our findings support a different scenario according to which deficits in action production/imitation are not paralleled by problems in action comprehension, as the MNM hypothesis predicts. In the study by Hamilton et al. (2007), children with ASD were asked to identify pictures of persons performing actions with the hands missing from the cartoon stimuli. Stimuli included both actions involving object use (e.g., a hand grabbing an iron) and symbolic actions (e.g., a soldier saluting an officer). The results showed that the ASD group performed significantly better than the control group of children with the same verbal mental age, suggesting that children with ASD do not have impaired gesture recognition. Smith and Bryson (1998) assessed the ability of children and adolescents with ASD to recognize nonsymbolic gestures, i.e., simple hand and finger postures and sequences, through a photo recognition task. The results revealed that the participants with ASD performed similarly to the control groups (children with receptive language delays and typically developing children) in the comprehension tasks. Interestingly, the authors found different results in the production tasks, as the ASD group performed poorly when asked to imitate those gestures. In a subsequent study, Smith and Bryson (2007) analyzed action imitation and comprehension of gestures with respect to two types of actions: social-communicative gestures and pantomimed actions with objects. Again, although the children and adolescents with ASD imitated less accurately than controls, the participants with ASD performed similarly to controls on the recognition and understanding of pantomimed actions.

In line with these findings, our results support the possibility that the MNM hypothesis does not account for the action representation in ASD. Indeed, they are in line with an alternative hypothesis according to which ASD involves a dissociation between action (i.e., gesture and pantomime) recognition and production/imitation (Hamilton et al., 2007; Smith and Bryson, 2007). This suggests two partly overlapping processing systems for gesture comprehension and gesture production. Interesting data supporting this alternative hypothesis come from the literature on patients with limb apraxia, which is an acquired deficit of gesture processing (Rothi et al., 1991; Cubelli et al., 2000; Bartolo et al., 2001). Patients with lesions in the parietal lobes have both recognition and imitation impairments, whereas patients with anterior lesions present deficits only in imitation, without any deficits in recognition. According to the dual-route model of gestural processing (Rothi et al., 1991), gestural representation relies on two independent routes: a lexical route responsible for the processing of meaningful gestures and a non-lexical route responsible for the processing of meaningless gestures. Accordingly, the lexical route is taken to support recognition, identification, and production of meaningful gestures, while the non-lexical route, which is a visuomotor conversion mechanism, should uphold the reproduction of all gestures including meaningless ones (Bartolo et al., 2001). On this model, it is possible for the lexical route to be selectively impaired, whereby the intact ability to recognize and identify a meaningful gesture *via* the action semantic system is accompanied by the inability to reproduce it. Instead, a malfunction in the visuomotor conversion mechanism might give rise to a selective production deficit limited to meaningless gestures. The dual-route model has been supported by research showing that individuals with ASD imitate meaningful actions more readily than meaningless ones (Rogers et al., 1996; Williams et al., 2004; Gizzonio et al., 2015). Furthermore, according to Williams et al. (2004), adding meaning to gesture facilitated performance in people with ASD.

The second major result of the current study concerns the presence of group-related differences across the conditions. The children with ASD who watched pantomimes in the informatively leaner condition performed worse than the children (both with ASD and TD) who were exposed to pantomimes in the informatively richer condition. Indeed, to understand the events presented in the informatively leaner condition, the children needed to imagine a piece of the missing information, whereas in the case of the informatively richer condition, the imaginative load was lower, because both the agent and the patient/beneficiary were pantomimically represented. This may have considerably affected the performance of the participants with ASD, who – as has often been reported – have a reduced capacity for imagination (e.g., Craig and Baron-Cohen, 1999; Crespi et al., 2016). Furthermore, the analysis of the different conditions revealed a positive correlation between a measure of working memory, i.e., the Forward Digit Span scores, and the pantomime score. This finding is in line with previous research (Bartolo et al., 2003; Villarreal et al., 2008) suggesting an important role of working memory in processing pantomimes. Therefore, the poorer performance of children with ASD in the informatively leaner condition may be also the result of their poorer working memory.

In addition to the involvement of working memory in the processing of pantomime, our findings suggest that the decreased ability to recognize pantomime by ASD individuals may at least partly result from a methodological bias regarding the poor information content of the delivered stimuli. This methodological bias is related to the narrow definition of pantomime adopted by previous studies (e.g., Ham et al., 2011; Cossu et al., 2012). Therefore, the current findings suggest the need to assume a broader definition of pantomime in future studies focusing on pantomime understanding in individuals with ASD. Indeed, when individuals with ASD are asked to interpret pantomimic events rather than individual imitated actions, they perform similarly to children with typical development. Furthermore, when the pantomimic events are represented in an informatively richer way, their performance (as well as that of children with typical development) significantly improves. This is a new aspect on the topic that is worth being investigated in future research.

## Conclusion

Our study explored the ability of a cohort of children with high-functioning ASD to understand pantomimic events. While in previous studies pantomime was conceived of as the mere execution of hand movements with or without object use (for transitive or intransitive actions, respectively), in this study we have adopted a richer definition of pantomime to differentiate it from other motor-visual communicative behaviors. Understood as a way of representing events by coordinated movements of the whole body, pantomime becomes a valuable tool for a broader understanding of the motor representations of actions in individuals with ASD. Therefore, our results showed that: (1) children with ASD generally managed to understand pantomimic events on a par with their typically developing peers; (2) the information content of pantomime – lean versus rich – influenced their performance, with the richer pantomimic events being easier to understand than the leaner ones.

## Ethics Statement

The study was approved by the Bambino Gesù Children’s Hospital Ethics Committee. Parents released their written informed consent to the participation of their children to the study and to the treatment of the data.

## Author Contributions

IA planned the study, adapted the original tests, administered the tasks, contributed to the interpretation of the data, and wrote the paper. FF planned the study, adapted the original tests, supervised the recruitment of the participants, and contributed to the interpretation of the data and to the writing of the Introduction and the Discussion. AC planned the study, adapted the original tests, administered the tasks, and contributed to the interpretation of the data and to the writing of the Introduction and the Discussion. SW contributed to planning the study and preparing the stimulus, and to the writing of the Introduction. PŻ contributed to planning the study, to the interpretation of the data, and to the writing of the Introduction. VD administered the tasks and contributed to the interpretation of the data. AM ran the statistics, contributed to the Results section, to the interpretation of the data, and the final version of the manuscript. RM contributed to the recruitment of the participants, administered the tasks, and contributed to the interpretation of the data. LC contributed to the recruitment of the participants. SV supervised the recruitment of the participants and the administration of the tasks. GV supervised the recruitment of the participants and the administration of the tasks and contributed to the interpretation of the data.

### Conflict of Interest Statement

The authors declare that the research was conducted in the absence of any commercial or financial relationships that could be construed as a potential conflict of interest.
